# A Bayesian framework for estimating the incremental value of a diagnostic test in the absence of a gold standard

**DOI:** 10.1186/1471-2288-14-67

**Published:** 2014-05-15

**Authors:** Daphne I Ling, Madhukar Pai, Ian Schiller, Nandini Dendukuri

**Affiliations:** 1 Department of Epidemiology and Biostatistics, McGill University, 1020 Pine Ave West, Montreal H3A 1A2, QC, Canada; 2 Division of Clinical Epidemiology, McGill University Health Centre–Research Institute, 687 Pine Avenue West, Room R4.09, Montreal H3A 1A1, QC, Canada

**Keywords:** Area under the curve, Bayesian estimation, Incremental value, Informative priors, Integrated discrimination improvement, Imperfect diagnostic tests, Latent class models, Tuberculosis

## Abstract

**Background:**

The absence of a gold standard, i.e., a diagnostic reference standard having perfect sensitivity and specificity, is a common problem in clinical practice and in diagnostic research studies. There is a need for methods to estimate the incremental value of a new, imperfect test in this context.

**Methods:**

We use a Bayesian approach to estimate the probability of the unknown disease status via a latent class model and extend two commonly-used measures of incremental value based on predictive values [difference in the area under the ROC curve (AUC) and integrated discrimination improvement (IDI)] to the context where no gold standard exists. The methods are illustrated using simulated data and applied to the problem of estimating the incremental value of a novel interferon-gamma release assay (IGRA) over the tuberculin skin test (TST) for latent tuberculosis (TB) screening. We also show how to estimate the incremental value of IGRAs when decisions are based on observed test results rather than predictive values.

**Results:**

We showed that the incremental value is greatest when both sensitivity and specificity of the new test are better and that conditional dependence between the tests reduces the incremental value. The incremental value of the IGRA depends on the sensitivity and specificity of the TST, as well as the prevalence of latent TB, and may thus vary in different populations.

**Conclusions:**

Even in the absence of a gold standard, incremental value statistics may be estimated and can aid decisions about the practical value of a new diagnostic test.

## Background

### Incremental value of a diagnostic test

The literature on diagnostic test evaluation has centered on estimation of sensitivity and specificity, measures that do not directly convey the clinical impact of a given test [1-3]. The added value of a test will depend on how much information is already available from the diagnostic work-up and whether the test result actually changes clinical decisions. The development of methods for evaluation of the incremental value of new tests or biomarkers is thus an active area of biostatistical research [4].

Evaluation of the incremental value of a new test typically involves comparing prediction models of the outcome of interest (measured by a gold standard), with and without the new test as a covariate. The difference between the area under the receiver operating characteristic curve (AUC), or the C-statistic, for the 2 models is the most familiar statistic for estimating incremental value [5]. The AUC measures the discrimination of a model, or its ability to distinguish between individuals with and without the outcome. One criticism of the AUC has been that it changes only slightly, even when effect measures such as the odds ratio suggest that a predictor is strongly associated with the outcome [6]. Another criticism is that the AUC has no direct clinical interpretation for individual patients. This has led to work on comparing predictive models in terms of the number of patients who are reclassified by adding a new test to an existing model.

Pencina and colleagues proposed 2 measures for the net increase in patients who are appropriately classified, i.e. higher predicted probabilities for patients with the outcome and lower probabilities for those without the outcome [7]. They defined the net reclassification improvement (NRI) as the increase in the proportion of patients who are accurately reclassified by the new versus the old model into pre-defined risk categories. They also proposed the integrated discrimination improvement (IDI) as a continuous version of the NRI across all possible risk thresholds from 0 to 1. The IDI is defined as the sum of the average increase in predicted probability among patients with the outcome and the average decrease in probability among patients without the outcome. Pepe and colleagues have also shown that the IDI is equivalent to the change in R^2^ for logistic regression [8].

### Evaluation of diagnostic tests in the absence of a gold standard

Both the AUC and IDI rely on the availability of information on the final outcome and assume that the true disease status can be determined with certainty (i.e., no misclassification). This assumption is not met for many diseases for which there is no gold standard, i.e., no diagnostic reference standard having perfect sensitivity and perfect specificity. Several approaches have been described for estimating disease prevalence and evaluating diagnostic accuracy in the absence of a gold standard [9]. Among these approaches, latent class models provide a more realistic interpretation of the problem by treating both the index and reference tests as imperfect [10,11].

In this article, we describe 2 ways in which these models can be used to estimate the incremental value of a new test compared to an imperfect reference test: 1) estimating the improvement in predicted probabilities using the AUC or IDI statistics 2) estimating the increase in correctly-classified patients using decisions rules based on observed test results. We illustrate our methods using simulated data and an application to estimating the incremental value of a diagnostic test for latent tuberculosis infection (LTBI). For diseases, such as LTBI, that have no clinically-relevant thresholds, it has been suggested that the IDI is more meaningful than the NRI [7,12]. Thus, our work focuses on the IDI and AUC difference but not the NRI statistic.

### Diagnosis of latent TB infection

TB is a leading cause of morbidity and mortality in the developing world [13]. LTBI can potentially develop into active disease without adequate preventive therapy. Unlike active TB (which can be detected with high accuracy using culture), LTBI has no gold standard. Until recently, the tuberculin skin test (TST) was the only screening test for LTBI. However, the TST suffers from imperfect sensitivity and specificity [14,15]. Interferon-gamma release assays (IGRAs), such as the QuantiFERON-TB Gold In-Tube (QFT), are now available and use antigens that are more specific to *M. tuberculosis* than the TST. Several meta-analyses show that the sensitivity of IGRAs is at least as good as the TST [16-18]. While the specificity of TST varies depending on when and how many BCG vaccines are given, the specificity of IGRAs is consistently high regardless of BCG vaccination [16-18]. Thus, a relevant question is whether IGRAs have any incremental value over the TST at the time of diagnosis in order to initiate preventive therapy, while using an approach that adjusts for the lack of a gold standard for LTBI.

## Methods

### Model for assessing incremental value without a gold standard

The observed data may be described by a latent class model which assumes that the standard test (T_1_) and new test (T_2_) are imperfect measures of an underlying latent variable D, or true disease status. Both tests and the disease status are assumed to be dichotomous, positive (+) or negative (-) based on standard cut-offs. The observed data follow a multinomial distribution where each probability of the 4 combinations of 2 tests can be expressed in terms of the sensitivity and specificity of both tests and the prevalence. Furthermore, each probability is a mixture of patients who are D + and D-:

(1) $$\begin{array}{l} P\left(T_{1}+,T_{2}+\right)=\pi \left(\mathrm{sen}s_{1}\mathrm{sen}s_{2}\right)+\left(1-\pi \right)\left(\left(1-\mathrm{spe}c_{1}\right)\left(1-\mathrm{spe}c_{2}\right)\right)\\ P\left(T_{1}+,T_{2}-\right)=\pi \left(\mathrm{sen}s_{1}\left(1-\mathrm{sen}s_{2}\right)\right)+\left(1-\pi \right)\left(\left(1-\mathrm{spe}c_{1}\right)\mathrm{spe}c_{2}\right)\\ P\left(T_{1}-,T_{2}+\right)=\pi \left(\left(1-\mathrm{sen}s_{1}\right)\mathrm{sen}s_{2}\right)+\left(1-\pi \right)\left(\mathrm{spe}c_{1}\left(1-\mathrm{spe}c_{2}\right)\right)\\ P\left(T_{1}-,T_{2}-\right)=\pi \left(\left(1-\mathrm{sen}s_{1}\right)\left(1-\mathrm{sen}s_{2}\right)\right)+\left(1-\pi \right)\left(\mathrm{spe}c_{1}\mathrm{spe}c_{2}\right), \end{array}$$

where π = P(D+) or the prevalence of disease, *sens*_j_ = P(T_j_ + |D+) or sensitivity of the j^th^ test (j = 1,2) and *spec*_j_ = P(T_j_-|D-) or specificity of the j^th^ test.

The latent class model in Equation (1) is non-identifiable due to the number of unknown parameters (5, i.e., sensitivity and specificity of both tests and prevalence) exceeding the degrees of freedom (3, i.e., possible test combinations - 1). This model can be estimated using a Bayesian approach with informative priors on at least 2 parameters (5 unknown parameters - 3 degrees of freedom) [10,19]. The prior information is combined with the observed data to obtain a joint posterior distribution. A sample from the posterior distribution can be drawn using Markov Chain Monte Carlo methods such as the Gibbs sampler [20]. To perform a Bayesian analysis, prior information on sensitivity and specificity must be expressed as probability distributions, such as the Beta distribution. Parameters for which no prior information is available may follow objective prior distributions, such as the Uniform distribution that assigns equal weight to all possible values.

### Estimation of incremental value

Let P(D + |T1, T2) denote the positive predictive probability given the results of T1 and T2, and let P(D+|T1) denote the positive predictive probability given T1 alone. Following Pencina *et al*. [7], we define the IDI as the difference of the differences between the expected (E) positive predictive probabilities with and without the new test, conditional on D + and D-:

(2) $$\begin{array}{l} \mathrm{IDI}=E\left(P\left(D+|T_{1},T_{2}\right)|D+\right)-E\left(P\left(D+|T_{1}\right)|D+\right)\\ \;-\left[E\left(P\left(D+|T_{1},T_{2}\right)|D-\right)-E\left(P\left(D+|T_{1}\right)|D-\right)\right]\\ \;=\sum_{u,v=-}^{+}P\left(D+|T_{1}=u,T_{2}=v\right)\\ \;P\left(T_{1}=u,T_{2}=v|D+\right)\\ \;-\sum_{u=-}^{+}P\left(D+|T_{1}=u\right)P\left(T_{1}=u|D+\right)\\ \;+[\sum_{u,v=-}^{+}P\left(D-|T_{1}=u,T_{2}=v\right)\\ \;P\left(T_{1}=u,T_{2}=v|D-\right)\\ \;-\sum_{u=-}^{+}P\left(D-|T_{1}=u\right)P\left(T_{1}=u|D-\right)] \end{array}$$

In the original definition by Pencina et al. [7], the predicted probabilities were derived from separate models--the old model based on T1 alone and the new model based on both T1 and T2. In the absence of a gold standard, the true disease status is unknown and must be estimated. We assumed that the latent class model for the joint results of T_1_ and T_2_ in Equation (1) provides the best estimate of an individual’s disease status, under the assumption of conditional independence. All predicted probabilities, whether conditional on T_1_ and T_2_ or T_1_ alone, were derived from this model. Furthermore, all the probabilities in Equation (2) can be expressed as functions of the sensitivity, specificity and prevalence estimates from the latent class model. For example,

$$\begin{array}{l} P\left(T_{1}+,T_{2}-|D+\right)=\mathrm{sen}s_{1}\left(1-\mathrm{sen}s_{2}\right)\;\mathrm{and}\\ P\left(D+|T_{1}+,T_{2}-\right)=\frac{\pi \mathrm{sen}s_{1}\left(1-\mathrm{sen}s_{2}\right)}{\pi \mathrm{sen}s_{1}\left(1-\mathrm{sen}s_{2}\right)+\left(1-\pi \right)\left(1-\mathrm{spe}c_{1}\right)\mathrm{spe}c_{2}}. \end{array}$$

The predictive values above can also be used to calculate the AUC. It may be calculated as the Wilcoxon rank sum statistic comparing predictive values in the groups D + and D- as follows [5]:

(3) $$\mathrm{AUC}=\frac{R_{D+}-\frac{N_{D+}\left(N_{D+}+1\right)}{2}}{N_{D+}\;N_{D-}},$$

where *R*_*D* +_ is the sum of the ranks of the positive predictive values calculated among the disease positive subjects and *N*_*D* +_ and *N*_*D* -_ are the number of disease positive and disease negative subjects, respectively. The AUC based on the probability conditional on T_1_ was subtracted from the AUC based on the probability conditional on T_1_ and T_2_ to obtain the AUC difference (AUC_diff_). A WinBUGS program for estimating the latent class model and the IDI and AUC_diff_ statistics appears in the Additional file 1: Table S1.

### Simulation study of model performance

We used the model in Equation (1) to generate simulated datasets to illustrate the change in IDI and AUC_diff_ when varying the sensitivity and specificity of T_2_. In all simulations, we assumed a sample size of N = 1000 and that both T_1_ and T_2_ were performed on all individuals. The sensitivity and specificity of T_1_ were set at 0.7 and 0.9, respectively; the prevalence was set at 0.3. We considered situations where the sensitivity (S) and/or specificity (C) of T_2_ was better (i.e., S_2_ = 0.8 and/or C_2_ = .95), worse (S_2_ = 0.6 and/or C_2_ = 0.8), or no different than T_1_. The true values of IDI and AUC_diff_ were calculated in each simulation setting using Equation (2) and Equation (3), respectively.

We generated 1000 datasets under each setting. We then fit the latent class model to the simulated datasets and estimated the AUC_diff_ and IDI statistics under each scenario. We used the results of the simulated datasets to estimate the frequentist properties of the AUC_diff_ and IDI statistics: average bias (i.e., the average difference between the true value and the posterior median across 1000 datasets), average coverage (i.e., the proportion of the 1000 datasets for which the posterior credible interval of a statistic included its true value) and average 95% posterior credible interval length.

As mentioned above, we need to specify at least 2 informative prior distributions for the model to be identifiable. We used 2 informative priors for the sensitivity and specificity of T_1_ (prior distribution ranging 0.7 ± 0.1 for sensitivity and 0.9 ± 0.05 for specificity) and uniform priors for the other parameters (i.e., sensitivity and specificity of T_2_ and prevalence). Prior information on the sensitivity and specificity of T_1_ was expressed as Beta(α,β) distributions by equating the midpoint of the range to the mean (μ) and one-quarter of the range to its standard deviation (σ) in order to obtain the alpha and beta parameters:

$$\alpha =-\frac{\mu \left(\sigma^{2}+\mu^{2}-\mu \right)}{\sigma^{2}}\mathrm{and}\;\beta =\frac{\left(\mu -1\right)\left(\sigma^{2}+\mu^{2}-\mu \right)}{\sigma^{2}}.$$

#### Impact of modeling conditional dependence

If the tests are positively correlated within the D + and D- groups, then their sensitivity and specificity may be overestimated or underestimated if this conditional dependence is ignored [21,22]. We carried out additional simulations assuming that the 2 tests are conditionally dependent, while retaining the same values for sensitivity and specificity as given above in the simulations involving the conditional independence model. The joint probabilities may be expressed as:

(4) $$\begin{array}{l} P\left(T_{1}+,T_{2}+\right)=\pi \left(\mathrm{sen}s_{1}\mathrm{sen}s_{2}+\mathrm{covs}\right)\\ \;+\left(1-\pi \right)\left(\left(1-\mathrm{spe}c_{1}\right)\left(1-\mathrm{spe}c_{2}\right)+\mathrm{covc}\right)\\ P\left(T_{1}+,T_{2}-\right)=\pi (\mathrm{sen}s_{1}(1-\mathrm{sen}s_{2})-\mathrm{covs})\\ \;+(1-\pi )((1-\mathrm{spe}c_{1})\mathrm{spe}c_{2}-\mathrm{covc}\\ )P\left(T_{1}-,T_{2}+\right)=\pi ((1-\mathrm{sen}s_{1})\mathrm{sen}s_{2}-\mathrm{covs})\\ \;+(1-\pi )(\mathrm{spe}c_{1}(1-\mathrm{spe}c_{2})-\mathrm{covc})\\ P\left(T_{1}-,T_{2}-\right)=\pi ((1-\mathrm{sen}s_{1})(1-\mathrm{sen}s_{2})+\mathrm{covs})\\ \;+(1-\pi )(\mathrm{spe}c_{1}\mathrm{spe}c_{2}+\mathrm{covc}), \end{array}$$

where *covs* and *covc* are the covariance between the tests in the D + and D- groups, respectively.

As described in Dendukuri and Joseph [21], these parameters were assumed to be bounded such that *covs* ~ dunif(0, min(*sens*_1_, *sens*_2_) - *sens*_1_*sens*_2_) and *covc* ~ dunif(0, min(*spec*_1_, *spec*_2_) - *spec*_1_*spec*_2_), allowing only for positive conditional dependence. To simulate data from Equation (4), we set *covs* and *covc* to the midpoint of their range to reflect a moderate degree of conditional dependence. Due to the addition of 2 unknown parameters, we need to provide informative priors on at least 4 parameters (7 unknown parameters - 3 degrees of freedom). Although the bounds on the covariance provide partial information, we estimated the model with additional informative priors on the sensitivity and specificity of T_2_. The corresponding Beta(α,β) prior distributions can be found in Additional file 2: Table S2.

#### Sensitivity to prior information

Non-identifiable latent class models are known to be heavily influenced by the subjective prior information used. While some may argue that it is impossible to study the consequences of prior misspecification because the prior information is subjectively defined for a given application, it is possible to study the impact of prior misspecification in a limited way in a simulated setting. We can expect that as the prior information moves away from the true values, the bias of the posterior estimates increases. However, this bias would also depend on the relative weight of the prior versus the data. Clearly, a weak prior distribution would cause less bias than a strong prior in the event that the prior is misspecified.

To examine the sensitivity of the IDI and AUC_diff_ statistics to prior information, we considered the following three types of prior misspecification that are likely to occur in practice: i) we replaced the range of prior information on the sensitivity and specificity of the standard test (T_1_) by point estimates that are equal to their true value. These would be very strong prior distributions, ii) we used point estimates of the sensitivity and specificity of T_1_ that were close to but not equal to their true values and iii) we used wide prior distributions on the sensitivity and specificity of T_1_ which covered the true value but were not centered on it. These would be weak prior distributions.

Situations (i) and (ii) are akin to assuming that the sensitivity and specificity of the standard test are perfectly known [23]--an assumption, which though hard to justify, is not uncommonly made in studies of accuracy or effectiveness of a new diagnostic test in the absence of a gold standard [19,24]. Situation (iii) reflects the consequences of misspecification of the relative importance of the true values when specifying an informative prior. It is more likely that the misspecified prior information is closer to the true values than being completely unrelated to the true values.

### Bayesian estimation

We used the BRugs package within R to fit the latent class model to each simulated dataset. To assess convergence, we ran 3 chains of the Gibbs sampler with different initial values. Convergence was checked by visual inspection of the history and density plots, and the Brooks-Gelman-Rubin statistic available within BRugs. We ran 50,000 iterations and dropped the first 5,000 burn-in iterations to report summary statistics based on 45,000 iterations (AUC was based on 5,000 iterations after model convergence). Median estimates from the posterior distribution are reported along with their 95% credible interval (CrI).

### Application to diagnosis of LTBI

We evaluated the incremental value of the QFT over TST in data from 2 published studies in India and Portugal, where both tests were performed simultaneously in healthcare workers with different BCG vaccination exposure. The TST has been shown to be less specific when the BCG vaccine is administered after infancy (e.g., during adolescence) or with multiple shots [18,25]. The Indian study consists of 719 healthcare workers, and 71% had a BCG vaccine scar [26]. Since the BCG vaccine is given once at birth in India, we expect the TST and QFT to perform similarly with respect to specificity [27]. In contrast, the Portuguese study consists of 1218 healthcare workers, and 70% had received ≥1 BCG vaccination after birth, which would lower the TST specificity [28].

We obtained prior information on the sensitivity and specificity of TST based on a previous meta-analysis [18]. The TST sensitivity ranged from 70% to 80%, while its specificity ranged from 96% to 99% for the Indian data. We expressed this as Beta(224.25, 74.75) and Beta(421.53, 10.81) distributions for the sensitivity and specificity, respectively. For the Portuguese data, the TST sensitivity also ranged from 70% to 80%, while its specificity ranged from 55% to 65%, corresponding to a Beta(229.8, 153.2) distribution.

Furthermore, we used Equation (4) to adjust for conditional dependence, since both TST and QFT measure cellular immune responses to *M. tuberculosis* antigens. We used informative priors for the sensitivity and specificity of QFT based on the same meta-analysis [18]. The sensitivity ranged from 70% to 80%, while the specificity ranged from 96% to 99% for both studies. These values were transformed into Beta(224.25, 74.75) and Beta(421.53, 10.81) distributions for the sensitivity and specificity, respectively. The prevalence and covariances were assumed to follow Uniform distributions. To study the sensitivity of the results to the form of the prior distribution, we replaced the Beta prior distributions by Uniform prior distributions with the same 95% CrI limits as those mentioned above. To study the sensitivity of the results to the prior distribution, we used a wider prior distribution whose 95% credible interval covered the lower and upper limits of the 95% confidence interval estimated for each individual study included in the meta-analysis.

### Decision rules for LTBI diagnosis based on observed data

In practice, the diagnosis of LTBI is based on observed test results rather than predicted probabilities from a latent class model [29]. Therefore, another way to view the incremental value of QFT is the increase in the number of individuals who are correctly classified (i.e., true positive and negative) within the D + and D- groups, compared to the classification based on TST alone. We compared the following decision rules based on one or both tests: 1) diagnose LTBI if TST + 2) diagnose LTBI if both TST + and QFT + 3) diagnose LTBI if either TST + or QFT+. The number of D + patients correctly classified by a decision rule is estimated as P(D + |rule+) multiplied by the number of patients who satisfy the rule. Similarly, the number of D- patients correctly classified is estimated as P(D-|rule-) multiplied by the number of patients who do not satisfy the rule.

As this study was conducted using simulated data and data from published articles, ethics approval was not required.

## Results

### Simulation study results

Table 1 illustrates the calculation of the IDI for the expected dataset (i.e., the dataset obtained by multiplying the probabilities in (1) by the sample size of 1000) for the case when sensitivity of T_2_ was higher than that of T_1_. The IDI can be decomposed into the incremental value among true-positive and true-negative patients. In this scenario, the predicted probability given both T_1_ and T_2_ increased among an estimated 17% of true-positive and 7% of true-negative patients, compared to the probability based on T_1_ alone. Thus, the overall estimate of the IDI was 24%.

**Table 1:** Step-by-step calculation of Integrated Discrimination Improvement (IDI) when Test 2 (T2) has higher sensitivity than Test 1 (T1)^*^

**T1, T2, D**	**Predicted probability conditional on T1 and T2 (P(D|T1,T2))**	**Predicted probability conditional on T1 alone (P(D|T1))**	**Difference**	**Weight (P(T1,T2|D))**	**Contribution to IDI (weight × difference)**
+ + +	0.96	0.75	0.21	0.56	0.12
+ - +	0.41	0.75	-0.34	0.14	-0.05
- + +	0.54	0.13	0.41	0.24	0.10
- - +	0.03	0.13	-0.1	0.06	-0.006
Incremental value among D + (Σweight × difference) = 0.17					
+ + -	0.04	0.25	-0.21	0.01	-0.002
+ - -	0.59	0.25	0.34	0.09	0.03
- + -	0.46	0.87	-0.41	0.09	-0.04
- - -	0.97	0.87	0.1	0.81	0.08
Incremental value among D- (Σweight × difference) = 0.07					
Overall incremental value = 0.24 (95% CrI: 0.10, 0.51)					

*D* = disease status.

*S_1_ = 0.7, C_1_ = 0.9, S_2_ = 0.8, C_2_ = 0.9, π = 0.3. Median values of each parameter are listed here only for illustration purposes. Calculations take into account the entire posterior distribution of each parameter.

The true values of the AUC_diff_ and IDI for all simulation scenarios are shown in Table 2 together with the estimated posterior median values (median, 2.5% and 97.5% quantiles) across 1000 datasets. The true incremental value was greatest when both sensitivity and specificity of T_2_ were higher than T_1_. As expected, the AUC_diff_ and IDI were close to 0 when T_2_ had no value, such that *sens*_*2*_ + *spec*_*2*_ = 0.7 + 0.3 = 1 (i.e., T_2_ was no better than a coin toss). Even when both sensitivity and specificity of T_2_ were lower than T_1_, there was some incremental value for T_2_. The incremental value was intermediate when either sensitivity or specificity of T_2_ was better or worse than T_1_. In all scenarios, the estimated values of the incremental value statistics were very close to the true values across the 1000 datasets. The frequentist properties of the IDI and AUC_diff_ statistics for each simulation scenario are given in Additional file 3: Table S3. In all scenarios, we find that the average coverage (i.e., the estimated probability that the posterior 95% credible interval for a certain statistic included its true value) exceeds 95% and the average bias (i.e., the difference between the true value and the posterior median) is low. We also confirmed convergence of the Gibbs sampler in each case (data not shown).

**Table 2:** True values and posterior estimates across 1000 simulated datasets of Area Under the Curve (AUC) and Integrated Discrimination Improvement (IDI) statistics obtained from latent class models assuming conditional independence (with S1=0.7, C1=0.9)

**Accuracy of T2**		**Parameter value**	**AUC for T1 and T2**	**AUC for T1**	**AUC difference**	**IDI in events**	**IDI in non events**	IDI*
1) Higher sensitivity	S_2_ = 80, C_2_ = 90	True	0.93	0.80	0.13	0.16	0.07	0.23
		Estimated	0.93 (0.91, 0.95)	0.80 (0.79, 0.80)	0.13 (0.11, 0.15)	0.17 (0.12, 0.22)	0.07 (0.05, 0.10)	0.25 (0.18, 0.32)
2) Higher specificity	S_2_ = 70, C_2_ = 95	True	0.92	0.80	0.12	0.16	0.07	0.23
		Estimated	0.94 (0.91. 0.96)	0.81 (0.80, 0.83)	0.12 (0.11, 0.14)	0.17 (0.12, 0.22)	0.07 (0.05, 0.09)	0.28 (0.22, 0.34)
3) Lower sensitivity	S_2_ = 60, C_2_ = 90	True	0.88	0.80	0.09	0.09	0.04	0.12
		Estimated	0.89 (0.87, 0.91)	0.80 (0.79, 0.80)	0.09 (0.07, 0.11)	0.10 (0.06, 0.14)	0.04 (0.03, 0.06)	0.14 (0.09, 0.20)
4) Lower specificity	S_2_ = 70, C_2_ = 80	True	0.88	0.80	0.09	0.07	0.03	0.10
		Estimated	0.89 (0.87, 0.91)	0.80 (0.79, 0.80)	0.09 (0.07, 0.11)	0.08 (0.05, 0.12)	0.03 (0.02, 0.05)	0.11 (0.07, 0.17)
5) Both better	S_2_ = 80, C_2_ = 95	True	0.94	0.80	0.14	0.21	0.09	0.30
		Estimated	0.94 (0.92, 0.96)	0.80 (0.80, 0.81)	0.14 (0.12, 0.17)	0.21 (0.16, 0.26)	0.09 (0.07, 0.11)	0.30 (0.23, 0.36)
6) Both worse	S_2_ = 60, C_2_ = 80	True	0.87	0.80	0.07	0.05	0.02	0.07
		Estimated	0.87 (0.85, 0.89)	0.80 (0.79, 0.80)	0.07 (0.05, 0.09)	0.05 (0.03, 0.08)	0.02 (0.01, 0.04)	0.07 (0.04, 0.12)
7) No better	S_2_ = 70, C_2_ = 90	True	0.90	0.80	0.10	0.12	0.05	0.17
		Estimated	0.91 (0.89, 0.94)	0.80 (0.79, 0.80)	0.11 (0.09, 0.14)	0.13 (0.09, 0.19)	0.06 (0.04, 0.08)	0.19 (0.13, 0.27)
8) No value	S_2_ = 70, C_2_ = 30	True	0.80	0.80	-0.00	-0.00	0.00	-0.00
		Estimated	0.81 (0.80, 0.82)	0.80 (0.79, 0.80)	0.01 (0.006, 0.02)	0.001 (0, 0.004)	<0.001 (0, 0.002)	0.002 (0.001, 0.006)

*T1* = test 1; *T2* = test 2; *S*_*2*_ = sensitivity of T2; *C*_*2*_ = specificity of T2.

*IDI is sum of IDI_events_ and IDI_non-events._

When T_2_ had higher sensitivity and specificity than T_1_ (S_2_ = 0.8, C_2_ = .95), the true AUC_diff_ was 0.14, which was only slightly better than when T_2_ had either higher sensitivity or higher specificity. In comparison, the magnitude of the true IDI was larger (IDI = 0.30) when T_2_ had higher sensitivity and specificity, compared to when T_2_ had either higher sensitivity (IDI = 0.25) or better specificity (IDI = 0.28). The IDI was lower when T_2_ had lower specificity of 0.8 (IDI = 0.11) compared to lower sensitivity of 0.6 (IDI = 0.14), due to the prevalence being less than 50%.

#### Impact of adjusting for conditional dependence

**Figure 1:**
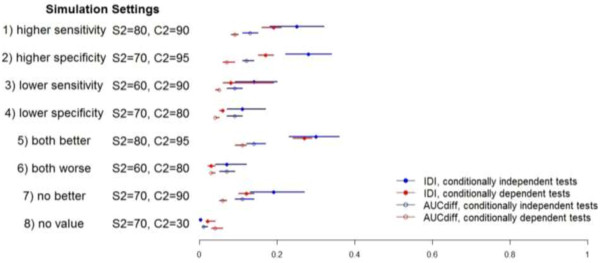
Incremental value of T2 over T1 based on simulation study (with S1 = 0.7, C1 = 0.9*). *Values plotted are median, 2.5% and 97.5% quantiles of posterior median values across 1000 datasets.

Table 3 shows the results for the simulations involving conditionally dependent tests: true values as well as a summary of the estimated values across 1000 simulated datasets. The incremental value was largest when T_2_ had better sensitivity and specificity than T_1_ and smallest when T_2_ was worse on both measures. When comparing this model to the one without adjustment for conditional dependence, the incremental value of T_2_ was generally lower (Figure 1; Tables 2 vs 3). Interestingly, the true value of both incremental value statistics suggested a small incremental value even when T_2_ was not useful: AUC = 0.04 and IDI = 0.02. This finding could be explained in part by the contribution of T_2_ being positively correlated with T_1_. Once again, the average bias was very small and the average coverage exceeded 95% across the 1000 simulated datasets in each scenario (Additional file 4: Table S4).

**Table 3:** True values and posterior estimates across 1000 simulated datasets of Area Under the Curve (AUC) and Integrated Discrimination Improvement (IDI) statistics obtained from latent class models assuming conditional dependence (with S1=0.7, C1=0.9)

**Accuracy of T2 compared to T1**		**Parameter value**	**AUC for T1 and T2**	**AUC for T1**	**AUC difference**	**IDI in events**	**IDI in non events**	IDI*
1) Higher sensitivity	S_2_ = 80, C_2_ = 90	True	0.88	0.80	0.09	0.10	0.04	0.15
		Estimated	0.90 (0.89, 0.90)	0.80 (0.80, 0.81)	0.09 (0.08, 0.10)	0.13 (0.11, 0.15)	0.06 (0.04, 0.07)	0.19 (0.16, 0.21)
2) Higher specificity	S_2_ = 70, C_2_ = 95	True	0.87	0.80	0.07	0.10	0.04	0.15
		Estimated	0.88 (0.87. 0.89)	0.80 (0.80, 0.81)	0.07 (0.06, 0.09)	0.12 (0.10, 0.14)	0.05 (0.04, 0.06)	0.17 (0.15, 0.19)
3) Lower sensitivity	S_2_ = 60, C_2_ = 90	True	0.84	0.80	0.04	0.03	0.01	0.04
		Estimated	0.85 (0.84, 0.86)	0.80 (0.80, 0.81)	0.05 (0.04, 0.05)	0.05 (0.04, 0.06)	0.02 (0.02, 0.03)	0.08 (0.06, 0.19)
4) Lower specificity	S_2_ = 70, C_2_ = 80	True	0.83	0.80	0.03	0.02	0.01	0.03
		Estimated	0.85 (0.84, 0.86)	0.80 (0.80, 0.81)	0.04 (0.04, 0.05)	0.04 (0.03, 0.04)	0.02 (0.01, 0.02)	0.06 (0.05, 0.06)
5) Both better	S_2_ = 80, C_2_ = 95	True	0.90	0.80	0.11	0.17	0.07	0.24
		Estimated	0.91 (0.90, 0.92)	0.80 (0.79, 0.81)	0.11 (0.09, 0.12)	0.19 (0.16, 0.21)	0.08 (0.07, 0.09)	0.27 (0.24, 0.29)
6) Both worse	S_2_ = 60, C_2_ = 80	True	0.82	0.80	0.02	0.01	0.00	0.01
		Estimated	0.84 (0.83, 0.84)	0.80 (0.80, 0.81)	0.03 (0.03, 0.04)	0.02 (0.02, 0.03)	0.01 (0.007, 0.01)	0.03 (0.02, 0.04)
7) No better	S_2_ = 70, C_2_ = 90	True	0.85	0.80	0.05	0.05	0.02	0.07
		Estimated	0.86 (0.85, 0.87)	0.80 (0.80, 0.81)	0.06 (0.05, 0.07)	0.08 (0.07, 0.10)	0.04 (0.03, 0.04)	0.12 (0.10, 0.14)
8) No value	S_2_ = 70, C_2_ = 30	True	0.84	0.80	0.04	0.01	0.01	0.02
		Estimated	0.84 (0.83, 0.86)	0.80 (0.79, 0.80)	0.04 (0.03, 0.06)	0.02 (0.01, 0.02)	0.01 (0.004, 0.01)	0.02 (0.02, 0.04)

*T1* = test 1; *T2* = test 2; *S*_*2*_ = sensitivity of *T2*; C_2_ = specificity of T2.

*IDI is sum of IDI_events_ and IDI_non-events_.

#### Sensitivity analysis in simulated scenarios

From Table 4 we can see that informative priors for S_1_ and C_1_ that are centered on the true value of a parameter would lead to the lowest bias and highest coverage, even compared to the case when the parameters are fixed at the true values. When S_1_ and C_1_ are fixed at values that are incorrect, though not far from the true values, the bias increases and coverage decreases sharply to 0. On the other hand, a wider prior that covers the true values but is not centered on them may have high coverage even though the bias is higher than the case when the prior is narrow and centered on the true values.

**Table 4:** **Sensitivity to prior distribution for the case when both sensitivity and specificity of the second test are better than the first test (true values are S**_**1**_ **= 0.7, C**_**1**_ **= 0.9)**

**Conditional Independence Model**						
**Prior information on T1 sensitivity and specificity**	**IDI (True value 0.3)**			**AUC**_**diff**_**(True value 0.14)**		
	**Bias**	**Length**	**Coverage**	**Bias**	**Length**	**Coverage**
Informative priors centered at true values*	0.001	0.43	1	-0.003	0.14	0.99
Degenerate priors at true values: S_1_ = 0.7, C_1_ = 0.9	0.006	0.19	0.95	-0.001	0.07	1
Degenerate priors, but not at true values: S_1_ = 0.8, C_1_ = 0.925	-0.17	0.08	0	-0.07	0.04	0
Informative priors covering but not centered on true values^†^ (centered on S_1_ = 0.8, C_1_ = 0.925)	-0.16	0.42	0.99	-0.07	0.15	1

*S1 ~ Beta(58.1, 24.9) (95% CrI 0.6, 0.8), C1 ~ Beta(128.7, 14.3) (95% CrI 0.85, 0.95).

^†^S1 ~ Beta(8.6, 1.4) (95% CrI 0.6, 0.99), C1 ~ Beta(38.1, 2.4) (95% CrI 0.85, 0.99).

It has been reported that ignoring conditional dependence between the tests when it exists will result in the latent class analysis providing biased estimates of the sensitivity, specificity and prevalence of both tests [21,22]. To study how ignoring conditional dependence will affect the incremental value statistics, we used the simulated datasets from the scenario where T_2_ had both improved sensitivity and specificity compared to T_1_ and the two tests are conditionally dependent. We found that incorrectly assuming conditional independence between the tests would result in over-estimating the incremental value. As seen in Table 3, the true values for this situation are AUC_diff_ =0.11 and IDI = 0.24. When ignoring the conditional dependence, the posterior median estimates were much higher across the 1000 simulated datasets: median AUC_diff_ = 0.15 [2.5% and 97.5% quantiles (0.13, 0.16)] and median IDI = 0.41 [2.5% and 97.5% quantiles (0.39, 0.47)].

#### Evaluation of QFT for LTBI

In the 2 published studies for the diagnosis of LTBI, the cross-tabulations of the test results were: TST + QFT + = 226, TST + QFT- = 62, TST-QFT + = 72, TST-QFT- = 359 for the Indian study [26] and TST + QFT + = 371, TST + QFT- = 532, TST-QFT + = 26, TST-QFT- = 289 for the Portuguese study [28]. Thus, the proportion of TST + QFT- results was higher in the Portuguese data. The estimates from the latent class model with conditional dependence appear in Table 5. For the Indian data, the AUC_diff_ was 0.08 (95% CrI: 0.06, 0.11), while the IDI was 0.23 (95% CrI: 0.16, 0.29). For the Portuguese data, these values were AUC_diff_ = 0.21 (95% CrI: 0.17, 0.25) and IDI = 0.40 (95% CrI: 0.29, 0.51). Thus, both AUC_diff_ and IDI indicated greater incremental value of the QFT in the Portuguese population where multiple BCG vaccinations compromise the TST specificity. In both studies, the IDI for events and nonevents are relatively equal, suggesting that the QFT changes the probabilities for individuals with and without LTBI to a similar extent.

**Table 5:** Median posterior estimates and 95% Credible Intervals (CrI) of latent class model parameters, Area Under the Curve (AUC) and Integrated Discrimination Improvement (IDI) statistics using data from applied examples

	**TST Sensitivity (95% CrI)**	**TST Specificity (95% CrI)**	**QFT Sensitivity (95% CrI)**	**QFT Specificity (95% CrI)**	**Prevalence (95% CrI)**	
India study (n = 719) [26]	0.74 (0.70, 0.78)	0.98 (0.96, 0.99)	0.76 (0.72, 0.80)	0.98 (0.96, 0.99)	0.53 (0.48, 0.58)	
Portugal study (n = 1218) [28]	0.84 (0.81, 0.87)	0.46 (0.42, 0.51)	0.69 (0.62, 0.75)	0.98 (0.97, 0.99)	0.47 (0.41, 0.55)	
	**AUC for TST and QFT (95% CrI)**	**AUC for TST (95% CrI)**	**AUC difference (95% CrI)**	**IDI in events (95% CrI)**	**IDI in non events (95% CrI)**	**IDI (95% CrI)**
India study (n = 719) [26]	0.94 (0.91, 0.97)	0.86 (0.83, 0.89)	0.08 (0.06, 0.11)	0.11 (0.07, 0.14)	0.12 (0.08, 0.16)	0.23 (0.16, 0.29)
Portugal study (n = 1218) [28]	0.86 (0.82, 0.89)	0.65 (0.61, 0.69)	0.21 (0.17, 0.25)	0.21 (0.13, 0.30)	0.19 (0.15, 0.22)	0.40 (0.29, 0.51)

#### Evaluating the sensitivity to the prior distribution

When using Uniform instead of Beta prior distributions, the results remain unchanged (data not shown). When using wider prior distributions, the posterior distributions for the sensitivity were wider and the median estimates of the specificities of both tests were lower in both the Indian and Portuguese data (Additional file 5: Table S5). Correspondingly, the median estimated incremental value statistics were all lower than those in Table 5. However, the credible intervals for these statistics (Additional file 5: Table S5) included the intervals in Table 5. Thus, our conclusion that the incremental value was higher for the Portuguese data remains unchanged.

#### Incremental value of QFT when using decision rules based on observed test results

In addition to the AUC_diff_ and IDI, the latent class model can be used to determine the incremental value based on the observed TST and QFT results by estimating the number of patients who are correctly classified as having or not having LTBI. As shown in Table 6, the TST + or QFT + decision rule would give the highest incremental value in the Indian data (9% increase in the number of correct diagnoses) mainly by increasing the proportion of true-positive patients compared to the diagnosis based on TST alone. The TST + or QFT + decision rule, however, would be similar to using the TST alone in the Portuguese data. Instead, the TST + and QFT + decision rule would result in the largest number of correctly-classified patients (21% increase in the number of correct diagnoses) mainly due to a decrease in the number of LTBI- patients who are false-positive. The difference in the preferred decision rule for the Indian and Portuguese data can be attributed to the different specificity of the TST test in these two groups. Nonetheless, the QFT test has incremental value over the TST in both populations. By calculating the incremental value as described, we can quantify precisely the expected impact of the difference in specificity for the two groups.

**Table 6:** Median number of patients classified correctly or misclassified under each decision rule for diagnosis of Latent Tuberculosis Infection (LTBI)

**Decision Rule**	**Classified correctly**			**Misclassified**			**Incremental value compared to LTBI if TST + (%)**
	**TP (%)**	**TN (%)**	**Total**	**FN (%)**	**FP (%)**	**Total**	
**India study (n = 719)**							
LTBI if TST+	280 (39)	331 (46)	611 (85)	100 (14)	8 (1)	108 (15)	-
LTBI if TST + and QFT+	223 (31)	335 (47)	558 (78)	158 (21)	3 (1)	161 (22)	-7
LTBI if TST + or QFT+	347 (48)	326 (46)	673 (94)	33 (4)	13 (2)	46 (6)	9
**Portugal study (n = 1218)**							
LTBI if TST+	504 (41)	245 (20)	749 (61)	70 (6)	399 (33)	469 (39)	-
LTBI if TST + and QFT+	361 (30)	634 (52)	995 (82)	213 (17)	10 (1)	223 (18)	21
LTBI if TST + or QFT+	528 (43)	243 (20)	771 (63)	46 (4)	401 (33)	447 (37)	2

*TP* = true positive; *FP* = false positive; *FN* = false negative; *TN* = true negative.

## Discussion

We have described how latent class models can provide information on the incremental value of a new diagnostic or screening test even in the absence of a gold standard test. Our simulations show that both the AUC_diff_ and IDI statistics can provide useful information on the incremental value in the absence of a gold standard. As in the case when a gold standard is present, the IDI statistic has a larger relative magnitude compared to the AUC_diff_ and can be interpreted as the average improvement in the predictive value. By considering different simulation settings for the new test’s accuracy, we found that the incremental value was greatest when both sensitivity and specificity of a new test were better than the standard test and that both incremental value statistics were close to zero when the new test was of no value. When adjusting for conditional dependence between tests, the incremental value of T_2_ was lower. When the model was mis-specified and ignored conditional dependence between the tests, both incremental value statistics were over-estimated as expected.

Bayesian estimation is particularly useful for latent class models that are non-identifiable due to insufficient degrees of freedom in the data, since it allows for the use of information external to the observed data. In our models, we used informative priors on the sensitivity and specificity of the standard test. As was the case in our motivating example of LTBI, evidence on these parameters can be obtained from the literature. One criticism of the latent class models we have used is their sensitivity to prior information. We believe that we have used the best available information on sensitivity and specificity of the TST and QFT tests resulting from a meta-analysis. Further, we carried out sensitivity analyses to other prior distributions. As we have shown, the Bayesian approach also provides credible intervals that have good coverage properties, unlike the limitations of the approximate frequentist intervals described previously for the IDI statistic [30].

We have argued that in the absence of a gold standard, all available test results are needed in the latent class model to provide the best estimate of the true disease status. Indeed, clinicians almost always rely on all available clinical information to make diagnostic decisions [24]. Alternative approaches to latent class analysis, including use of a composite reference standard or panel diagnosis, define a decision rule to definitively classify patients as disease positive or disease negative. Once such a definitive classification of disease status is obtained, methods for estimating incremental value in the presence of a gold standard may be used. The concern with this approach, of course, is that it may lead to reference standard bias [31]. In some situations, it may not be possible to implement these alternatives. In our motivating example, there were only two tests. Thus, it was not possible to define a composite reference standard. Moreover, if we used the simplistic approach of treating the older test as a gold standard, it would be equivalent to assuming that the new test has no incremental (or added) value. Hence, we feel a latent class approach is particularly valuable in this setting.

Since this is the first paper on the topic of estimating incremental value in the absence of a gold standard, we chose to focus on illustrating the concept in the simplest case involving only observed data from two diagnostic tests. We recognize that our model does not include patient characteristics (e.g., age) that may play a role in the diagnostic decision-making process, thereby limiting the variation in predicted probabilities. Further research is needed to extend latent class models to incorporate such covariates that could have an effect on the prevalence, sensitivity or specificity [32]. In addition, more complex models can be used when test results are continuous or there are more than 2 tests involved. Findings from previous work showing that increased sample size and an increase in informativeness of the prior distributions improve the precision of parameter estimates from a non-identifiable latent class model would also apply here [33], as all incremental statistics that we have described are functions of the prevalence, sensitivity and specificity parameters in the latent class model. In addition, more research is needed to examine the impact of the choice of a particular conditional dependence structure and the degree of conditional dependence on estimates of incremental value.

Another future direction would be estimating the more common NRI using plausible risk thresholds or even the category-free version [12]. In particular, our method may be able to address a recent criticism that the NRI cannot measure improvements in risk prediction at the population level [34], since the latent class model incorporates prevalence into the estimates. It should be mentioned that a number of recent articles have been critical of the IDI and NRI statistics [30,35]. In particular, it has been pointed out that they can be inflated for miscalibrated prediction models whereas the AUC may not be. This remains to be studied in the context when there is no gold standard.

An alternative approach to adding covariates to the model is to carry out a subgroup analysis. In our LTBI example, we estimated incremental value within subgroups defined by study setting. The QFT had different incremental value beyond the TST depending on the population and BCG vaccination policy. In low-risk groups, using the TST + and QFT + decision rule could help avoid unnecessary LTBI therapy. On the other hand, using the TST + or QFT + decision rule could help clinicians who are worried about missing LTBI cases in high-risk groups, such as HIV/AIDS patients and young children. Such reasoning has been used to support cost-effectiveness analyses of the TST and QFT for diagnosis of LTBI [36]. The Bayesian approach we propose is an improvement over such approaches since it takes into account the joint uncertainty in the sensitivity and specificity parameters of both tests [37].

Several national guidelines now exist on using IGRAs such as the QFT, and many low-incidence countries recommend a two-step process: if TST is positive then perform the QFT as a confirmatory test [29]. This approach is equivalent to the “diagnose LTBI if TST + and QFT+” rule in terms of incremental value but is cheaper since not all patients receive both tests. In fact, the “diagnose LTBI if QFT+” rule (data not shown) would give similar results compared to the TST + and QFT + rule in the Portuguese data. However, the QFT is sold as a commercial kit that is more expensive than the TST. Ultimately, the decision to implement a new test into practice will depend on many factors, including patient preferences, risk of complications and cost considerations.

## Conclusions

We have illustrated how to estimate incremental value in the absence of a gold standard test by relying on prior information on the sensitivity and specificity of one or both tests. Using point priors rather than prior ranges results in poor coverage and bias compared to using a wide prior, even if it is not centered on the true values. Further research is needed to develop methods for estimation of incremental value conditional on more tests and covariates.

## Competing interests

The authors declare that they have no competing interests.

## Author’s contributions

DL participated in the study design, performed the analysis and wrote the manuscript as first author. MP provided assistance with the data analysis and interpretation. IS performed additional analysis and provided interpretation of the results. ND designed the study, participated in the analysis and supervised the study. All authors read and approved the final manuscript.

## Pre-publication history

The pre-publication history for this paper can be accessed here:

http://www.biomedcentral.com/1471-2288/14/67/prepub

## Supplementary Material

Additional file 1: Table S1 WinBUGS program for estimating the latent class model, IDI and AUC_diff_ statistics. Click here for file

Additional file 2: Table S2 Priors for T2 in simulation study of the conditional dependence model. Click here for file

Additional file 3: Table S3 Average coverage, average bias and average length of 95% posterior credible intervals of AUC_diff_ and IDI statistics resulting from fitting conditional independence latent class model to 1000 simulated datasets. Click here for file

Additional file 4: Table S4 Average coverage, average bias and average length of 95% posterior credible intervals of AUC_diff_ and IDI statistics resulting from fitting conditional dependence latent class model to 1000 simulated datasets. Click here for file

Additional file 5: Table S5 Median posterior estimates and 95% credible intervals of parameters for latent class model and AUC_diff_ and IDI statistics using data from applied examples when using wider prior distributions. Click here for file
