# Supplementary material for: A Bayesian framework for estimating the incremental value of a diagnostic test in the absence of a gold standard
Source: BMC Med Res Methodol. 2014 May 15;14:67. doi: 10.1186/1471-2288-14-67 (PMC4077291; doi:10.1186/1471-2288-14-67)
Supplement: Additional file 1: Table S1 — WinBUGS program for estimating the latent class model, IDI and AUCdiff statistics. [file 1471-2288-14-67-S1.docx]

ADDITIONAL FILES FOR **“A Bayesian framework for estimating the incremental value of a diagnostic test in the absence of a gold standard”**

**A1.** WinBUGS program for estimating the latent class model, IDI and AUC_diff_ statistics

#==================

# model statements

#=================

model fixed;

{

#=====================================================================

# prior distributions of prevalence, sensitivities and specificities

#=====================================================================

pi~dbeta(alpha.pi,beta.pi);

sens1~dbeta(alpha.sens1,beta.sens1);

spec1~dbeta(alpha.spec1,beta.spec1);

sens2~dbeta(alpha.sens2,beta.sens2);

spec2~dbeta(alpha.spec2,beta.spec2);

#=======================================

# upper limits of covariance parameters

#=======================================

us<-min(sens1,sens2)-(sens1*sens2);

uc<-min(spec1,spec2)-(spec1*spec2);

#==============================================================

# prior distribution of transformed covariances on (0,1) range

#==============================================================

u.covs12~dbeta(alpha.covs12,beta.covs12);

u.covc12~dbeta(alpha.covc12,beta.covc12);

#==============================================

# adjustment of range of covariance parameters

# (REMOVE ZEROES IN THE FOLLOWING TWO LINES TO ADJUST FOR CONDITIONAL DEPENDENCE)

#==============================================

covs12<-0; #*u.covs12*us;

covc12<-0; #*u.covc12*uc;

#==============================================

# conditional correlation

#==============================================

corrs12<-covs12/sqrt(sens1*(1-sens1)*sens2*(1-sens2));

corrc12<-covc12/sqrt(spec1*(1-spec1)*spec2*(1-spec2));

#=============================================================

# Observed values of multinomial random variable measuring

# joint results of two dichotomous diagnostic tests

#============================================================

t12[1]<-n11;

t12[2]<-n10;

t12[3]<-n01;

t12[4]<-n00;

#======================================================================

# probabilities of observing different cross-classifications of two dichotomous diagnostic tests

#======================================================================

p12[1]<-pi*(sens1*sens2+covs12)+(1-pi)*((1-spec1)*(1-spec2)+covc12);

p12[2]<-pi*(sens1*(1-sens2)-covs12)+(1-pi)*((1-spec1)*spec2-covc12);

p12[3]<-pi*((1-sens1)*sens2-covs12)+(1-pi)*(spec1*(1-spec2)-covc12);

p12[4]<-pi*((1-sens1)*(1-sens2)+covs12)+(1-pi)*(spec1*spec2+covc12);

#==============================

# likelihood of observed data

#===============================

t12[1:4] ~ dmulti(p12[1:4],N)

#==============================

# new predicted value positive

#===============================

ppv.new[1]<-(pi*(sens1*sens2+covs12))/p12[1]

ppv.new[2]<-(pi*(sens1*(1-sens2)-covs12))/p12[2]

ppv.new[3]<-(pi*((1-sens1)*sens2-covs12))/p12[3]

ppv.new[4]<-(pi*((1-sens1)*(1-sens2)+covs12))/p12[4]

#==============================

# old predicted value positive

#===============================

ppv.old[1]<-(pi*sens1)/(pi*sens1+(1-pi)*(1-spec1))

ppv.old[2]<-(pi*sens1)/(pi*sens1+(1-pi)*(1-spec1))

ppv.old[3]<-(pi*(1-sens1))/(pi*(1-sens1)+(1-pi)*spec1)

ppv.old[4]<-(pi*(1-sens1))/(pi*(1-sens1)+(1-pi)*spec1)

#==============================

# IDI

#===============================

for(i in 1:4) {

idi.events1[i]<-(ppv.new[i]-ppv.old[i])*((ppv.new[i]*p12[i])/pi)

idi.nonevents1[i]<-(ppv.old[i]-ppv.new[i])*(((1-ppv.new[i])*p12[i])/(1-pi))

idi1[i]<-idi.events1[i]+idi.nonevents1[i]

}

idi.events<-sum(idi.events1[])

idi.nonevents<-sum(idi.nonevents1[])

idi<-sum(idi1[])

# ================================

# AUC and AUC_diff_

# ================================

for (j in 1:4) { npos[j]<-round(ppv.new[j]*t12[j]) }

n1<-sum(npos[])

n0<-N-n1

r.old[1]<-rank(ppv.old[],1)

r.old[2]<-rank(ppv.old[],3)

r.old1<-r.old[1]-1

r.old2<-r.old[2]-1

n1.old<-t12[r.old[1]]+t12[r.old1]

n2.old<-t12[r.old[2]]+t12[r.old2]

rank.sum.old<-((n1.old+1)/2)*(npos[r.old[1]]+npos[r.old1])+(n1.old+(n2.old+1)/2)*(npos[r.old[2]]+npos[r.old2])

r.new[1]<-rank(ppv.new[],1)

r.new[2]<-rank(ppv.new[],2)

r.new[3]<-rank(ppv.new[],3)

r.new[4]<-rank(ppv.new[],4)

n1.new<-t12[r.new[1]]

n2.new<-t12[r.new[2]]

n3.new<-t12[r.new[3]]

n4.new<-t12[r.new[4]]

rank.sum.new<-((n1.new+1)/2)*npos[r.new[1]]+(n1.new+(n2.new+1)/2)*npos[r.new[2]]+((n1.new+n2.new)+(n3.new+1)/2)*npos[r.new[3]]+((n1.new+n2.new+n3.new)+(n4.new+1)/2)*npos[r.new[4]]

auc.old<-(rank.sum.old-(n1*(n1+1)/2))/(n1*n0)

auc.new<-(rank.sum.new-(n1*(n1+1)/2))/(n1*n0)

auc.diff<-auc.new-auc.old

}
