# Supplementary material for: A Bayesian framework for estimating the incremental value of a diagnostic test in the absence of a gold standard
Source: BMC Med Res Methodol. 2014 May 15;14:67. doi: 10.1186/1471-2288-14-67 (PMC4077291; doi:10.1186/1471-2288-14-67)
Supplement: Additional file 2: Table S2 — Priors for T2 in simulation study of the conditional dependence model. [file 1471-2288-14-67-S2.docx]

**A2.** Priors for T2 in simulation study of the conditional dependence model

| **Scenario** | **Range** | **Beta(α,β)** |
| --- | --- | --- |
| 1) higher sensitivity | S_2_=(70, 90)  C_2_=(80, 100) | (50.4, 12.6)  (31.5, 3.5) |
| 2) higher specificity | S_2_=(60, 80)  C_2_=(90, 100) | (58.1, 24.9)  (71.25, 3.75) |
| 3) lower sensitivity | S_2_=(50, 70)  C_2_=(80, 100) | (57, 38)  (31.5, 3.5) |
| 4) lower specificity | S_2_=(60, 80)  C_2_=(70, 90) | (58.1, 24.9)  (50.4, 12.6) |
| 5) both sensitivity and specificity better | S_2_=(70, 90)  C_2_=(90, 100) | (50.4, 12.6)  (71.25, 3.75) |
| 6) both sensitivity and specificity worse | S_2_=(50, 70)  C_2_=(70, 90) | (57, 38)  (50.4, 12.6) |
| 7) no better | S_2_=(60, 80)  C_2_=(80, 100) | (58.1, 24.9)  (31.5, 3.5) |
| 8) no value | S_2_=(60, 80)  C_2_=(20, 40) | (58.1, 24.9)  (24.9, 58.1) |

S_2_ = sensitivity of T2; C_2_ = specificity of T2
