# Supplementary material for: A Bayesian framework for estimating the incremental value of a diagnostic test in the absence of a gold standard
Source: BMC Med Res Methodol. 2014 May 15;14:67. doi: 10.1186/1471-2288-14-67 (PMC4077291; doi:10.1186/1471-2288-14-67)
Supplement: Additional file 3: Table S3 — Average coverage, average bias and average length of 95% posterior credible intervals of AUCdiff and IDI statistics resulting from fitting conditional independence latent class model to 1000 simulated datasets. [file 1471-2288-14-67-S3.docx]

**A3**. Average coverage, average bias and average length of 95% posterior credible intervals of AUC_diff_ and IDI statistics resulting from fitting conditional independence latent class model to 1000 simulated datasets

| Accuracy of T2 compared to T1 | | **AUC_diff_** | | | **IDI** | | |
| --- | --- | --- | --- | --- | --- | --- | --- |
|  |  | Average Bias | Average Length | Average Coverage | Average Bias | Average Length | Average Coverage |
| 1) higher sensitivity | S_2_=80, C_2_=90 | 0.002 | 0.14 | 1.00 | 0.02 | 0.39 | 1.00 |
| 2) higher specificity | S_2_=70, C_2_=95 | 0.001 | 0.14 | 1.00 | 0.01 | 0.40 | 1.00 |
| 3) lower sensitivity | S_2_=60,  C_2_=90 | 0.004 | 0.12 | 1.00 | 0.02 | 0.29 | 1.00 |
| 4) lower specificity | S_2_=70,  C_2_=80 | 0.003 | 0.11 | 1.00 | 0.01 | 0.25 | 1.00 |
| 5) both better | S_2_=80, C_2_=95 | -0.003 | 0.14 | 1.00 | 0.001 | 0.43 | 1.00 |
| 6) both worse | S_2_=60, C_2_=80 | 0.002 | 0.09 | 1.00 | 0.01 | 0.18 | 1.00 |
| 7) no  better | S_2_=70, C_2_=90 | 0.008 | 0.13 | 1.00 | 0.02 | 0.36 | 1.00 |
| 8) no  value | S_2_=70,  C_2_=30 | 0.01 | 0.04 | 0.99 | 0.002 | 0.01 | 1.00 |
