# Supplementary material for: A Bayesian framework for estimating the incremental value of a diagnostic test in the absence of a gold standard
Source: BMC Med Res Methodol. 2014 May 15;14:67. doi: 10.1186/1471-2288-14-67 (PMC4077291; doi:10.1186/1471-2288-14-67)
Supplement: Additional file 5: Table S5 — Median posterior estimates and 95% credible intervals of parameters for latent class model and AUCdiff and IDI statistics using data from applied examples when using wider prior distributions. [file 1471-2288-14-67-S5.docx]

**A5.** Median posterior estimates and 95% credible intervals of parameters for latent class model and AUC_diff_ and IDI statistics using data from applied examples when using wider prior distributions^*^

|  | **TST Sensitivity**  **(95% CrI)** | **TST Specificity**  **(95% CrI)** | **QFT Sensitivity**  **(95% CrI)** | **QFT Specificity**  **(95% CrI)** | **Prevalence**  **(95% CrI)** | |
| --- | --- | --- | --- | --- | --- | --- |
| India study (n=719) [26] | 0.74  (0.54, 0.93) | 0.89  (0.78, 0.97) | 0.75  (0.55, 0.92) | 0.88  (0.76, 0.97) | 0.46  (0.33, 0.67) | |
| Portugal study (n=1218) [28] | 0.87  (0.67, 0.96) | 0.33  (0.24, 0.60) | 0.67  (0.39, 0.90) | 0.87  (0.71, 0.96) | 0.36  (0.08, 0.73) | |
|  | **AUC for TST and QFT**  **(95% CrI)** | **AUC for TST**  **(95% CrI)** | **AUC difference**  **(95% CrI)** | **IDI in events**  **(95% CrI)** | **IDI in**  **non events**  **(95% CrI)** | **IDI**  **(95% CrI)** |
| India study (n=719) [26] | 0.90  (0.76, 0.96) | 0.81  (0.69, 0.93) | 0.07  (0.01, 0.17) | 0.06  (0.002, 0.22) | 0.06  (0.001, 0.17) | 0.12  (0.003, 0.39) |
| Portugal study (n=1218) [28] | 0.81  (0.66, 0.91) | 0.61  (0.50, 0.72) | 0.19  (0.05, 0.36) | 0.09  (0.004, 0.21) | 0.16  (0.01, 0.40) | 0.25  (0.01, 0.60) |

^*^ Beta prior distribution parameters (and corresponding 95% credible intervals) were as follows:

For India: S1 ~ Beta(5.27, 2.43) (95% CrI 0.37, 0.99), C1 ~ Beta(27.91, 3.45) (95% CrI 0.78, 0.99), S2 ~ Beta(9.36, 3.03)(95% CrI 0.52, 0.99), C2 ~ Beta(23.63, 3.38)(95% CrI 0.75, 0.99);

For Portugal: S1 ~ Beta(5.27, 2.43)(95% CrI 0.37, 1), C1 ~ Beta(6.00, 4.72)(95% CrI 0.27, 0.85), S2 ~ Beta(9.36, 3.04)(95% CrI 0.52, 0.99), C2 ~ Beta(16.07, 3.18)(95% CrI 0.67, 0.99)
